# Research on innovative design of Nuo mask based on Memetics-AHP-Thematic analysis-shape grammar

**DOI:** 10.1371/journal.pone.0326630

**Published:** 2025-07-14

**Authors:** Axiang Yang, Leqiong Wang, Wangqun Xiao, Long Chen, Xinye Liao

**Affiliations:** Industrial Design Engineering, Academy of Arts and Design, Anhui University of Technology, Maanshan, China; University of Opole, POLAND

## Abstract

The study seeks to investigate the cultural factors of Chizhou Nuo masks and their strategic application strategies in contemporary design, by compiling the Chizhou Nuo cultural literature, integrating memetics, identifying the cultural factors of the Chizhou Nuo masks, and then developing the genetic mapping of the Chizhou Nuo mask culture. Simultaneously, the analytic hierarchy process (AHP) is employed to objectively allocate weights to design considerations, ascertain the significance of each cultural factor, and prioritize their value, thereby filtering the necessary design elements. Subsequently, thematic analysis to employed to examine the interview data, yielding an indicator of consumer demand. The shape grammar was ultimately utilized to produce a design product that adheres to modern aesthetic criteria. The fuzzy comprehensive evaluation (FCE) method is used to evaluate the design scheme, select the optimal design scheme, and guide further design optimization. This study investigates a novel paradigm for the design and evaluation of Chizhou Nuo mask cultural and creative items utilizing memetics, AHP, thematic analysis, shape grammar, and FCE. The model’s practicality is validated through the design cases of Chizhou Nuo mask graphic design. It offers novel theoretical and practical direction for the designers of future intangible cultural and creative products. Furthermore, it offers a novel avenue for the preservation and advancement of intangible cultural heritage.

## 1. Introduction

In the global context, the safeguarding of intangible cultural heritage has emerged as a universal consensus. Nuo opera is a significant cultural manifestation in China’s extensive history, characterized by dispersion, aggregation, mutation, and emergence. Despite apparent differences, these cultural varieties share an underlying commonality. As one of China’s oldest and most comprehensive folk rituals, Nuo opera embodies a rich array of national characteristics and serves as a symbol of Chinese culture [1]. The Chizhou Nuo culture encompasses various disciplines, including theater studies, anthropology, sociology, history, religious studies, artology, archaeology, and folklore. And it always maintains a simple, rugged, and original appearance, bringing a unique visual experience to the viewer. Masks are a fundamental component of Nuo activities and serve as its focal point. In the realm of Nuo activities, the reverence for the mask is remarkably profound; it is forbidden to bathe or fast in proximity to the mask, and it cannot be deemed a Nuo mask without illumination [2]. Masks in Nuo opera are not constructed from masonry but are instead crafted through make-up, representing an exquisite form of handicraft. These masks enhance the visual aesthetic for scholars, who utilize Nuo masks to explore the underlying humanistic significance of Nuo culture, its cultural values, stylistic colors, and visual artistry, thereby offering a novel direction for the design of Nuo derivatives. Currently, the majority of scholars researching Nuo opera concentrate on the academic dimensions of Nuo culture. However, the examination of Nuo masks, particularly on their heritage and sustainable growth, remains insufficient. This study aims to comprehensively inherit Nuo culture while fostering the local economy in Chizhou by integrating memetics, extraction techniques, and creative design methods that reflect regional culture characteristics into product design. The objective is to achieve aesthetic appeal alongside the preservation of cultural heritage, thereby exploring the innovative design and sustainable development pathways for Nuo culture and its regional adaptation in Chizhou.

## 2. Literature review

Domestic researchers examine Chizhou Nuo culture from several viewpoints, including folklore, artology, folk crafts, and aesthetics, focusing on its connotation, inheritance, protection, and development. Foreign scholars, including Japanese researchers Seiji Ito, Oki Yasushi, Hirota Ritsuko, Suzuki Masataka, and Akiko Inada, along with Korean scholars Sookyung Oh, Choi Yong Soo [3–9] and so on, examine the performance of Chinese Nuo opera, its cultural connotations, and the historical significance of theater from a comparative perspective. Domestic researchers examine Nuo masks to explore the human significance and cultural values they embody, as well as their stylistic colors and visual artistry, so offering a novel perspective for the construction of Nuo culture derivatives. Scholars Lin G X, Xiong A L [10–11], and others illustrate the craftsmanship, aesthetic features, and derivative worth of Nuo masks. Nevertheless, there is a paucity of research around the concept of industrial design. The examination of Nuo masks hides cultural influences in product design.

Design elements must be selected for their high recognizability and capacity to serve as a visual symbol that stimulates user perception and imagination. Identifying design characteristics beforehand can unify the design style and improve the object’s recognizability, identity, and appeal [12]. This study will identify the cultural characteristics of the Nuo mask for the design of cultural and creative products. During the factor establishment phase, the majority of extant research begins by extracting cultural elements from traditional culture, followed by the redesign of factors that align with contemporary aesthetics. Scholars Chen X employs Kansei Engineering and Statistics, utilizing user satisfaction analysis to identify the factors of the Shadow Puppets for design purposes [13]; scholar Gou B C applies the principles of genetic engineering to extract the culture genes of Banpo pottery for design applications [14]; scholar Jiang F utilizes the analytic hierarchy process to examine the relationships and gradations among the factors of the Hakka culture in Gannan, constructing a hierarchical structure model of enclosure culture to rank these factors by importance for modern product design [15]. The extant literature on traditional cultural product design offers innovative concepts but is deficient in research regarding consumer desire. This article examines the design choices of the cultural and creative product informed by user needs prior to the product design of the Chizhou Nuo mask.

During the user analysis phase, the majority of current research employs interviews and questionnaires to gather user demand metrics. The researchers classify, summarize, and generalize the interview data. This technique is subjective and devoid of the principles of qualitative research theory. This research establishes a theoretical framework for methodically and scientifically analyzing user demands derived from semi-structured interviews. Textual Thematic Analysis is a rigorous qualitative research methodology that involves the methodical collection of data, identification of fundamental concepts that encapsulate the essence of phenomena, and the formulation of pertinent substantive hypotheses through the interrelation of these concepts. Its benefit is in its ability to methodically examine, refine, and summarize the raw data. For example, Zhu Q X and other scholars employed thematic analysis to examine 27 articles from three prominent databases, identifying four principal themes related to souvenirs and cultural heritage [16]; Cui X and other associates utilized the Python programming language to develop the LDA model, performing thematic mining and evolutionary analysis of news reports from the archive’s official website and the NRLC website, and through a comparison, subsequently analyzing salient issues regarding the archive’s non-heritage preservation [17]; Amy Shirong Lu and other scholars conducted interviews with children aged 8–12, applying exploratory thematic analysis to digitally record, transcribe, and analyze the discussions to ascertain their preferences [18]. The thematic analysis examines and classifies textual content to discern and comprehend particular themes or concerns. This work employed thematic analysis to examine the interview data in order to ascertain consumers’ preferences regarding the design requirements for the Chizhou Nuo mask.

In the design evaluation process, numerous studies have utilized the Analytic Hierarchy Process (AHP) and Fuzzy Comprehensive Evaluation (FCE) methods, individually or in combination. The AHP is widely favored by experts and researchers for assessing complex issues with multiple objectives and criteria due to its robust theoretical foundation, structured methodology, and ease of application. It effectively combines qualitative and quantitative approaches, offering advantages in organization and systematic analysis. Fuzzy Comprehensive Evaluation can be applied to assess and optimize design options, helping to identify the best solution and refine it for improvement. In product concept development, AHP is used to break down qualitative design decision factors into hierarchical layers. It allows for the computation of indicator weights through quantitative analysis, systematically ranking these indicators. As a result, AHP and FCE are frequently employed in design decision-making. For instance, Liu Mingbin and other researchers used AHP to derive a comprehensive ranking of factors relevant to restaurant chair design, providing designers with critical design insights and quantifiable metrics during the initial design phase. They later utilized fuzzy hierarchical analysis to perform quantitative evaluations of three design schemes, ultimately selecting the optimal solution. Similarly [19]; Sun Yang and colleagues applied AHP to determine indicator weights and subsequently employed Fuzzy Comprehensive Evaluation to evaluate unmanned ground vehicles [20]. Therefore, this paper adopts AHP and Fuzzy Comprehensive Evaluation methods to score design schemes, aiming to select the optimal solution that aligns with consumer needs and preferences.

The research mentioned above findings indicate the application of shape grammar in the design practice of Chizhou Nuo masks. This study introduces a novel integrated approach combining cultural elements, AHP, thematic analysis, shape grammar and FCE for the design of traditional cultural products, with the objective of enhancing the objectivity and rationality of the design process. The method will offer theoretical guidance for future designers and aid in design choices. Consequently, it fosters the creation of contemporary cultural items to satisfy consumer demands more effectively.

## 3. Methods

### 3.1. Theoretical overview

**Memetics.** In 1976, academic Richard Dawkins introduced the concept of Mimeme in his book 《The Selfish Gene》, likening the Meme to a genetic element that facilitates the dissemination of culture or imitation at the level of the person [21]. Cultural genes are a fundamental element in the regional cultural design, demonstrating a distinctive mode of expression [22]. Through the systematic excavation and sorting of excellent traditional cultural genes, a gene map was created to extract typical design factors, which were applied to the later design practice.

**Analytic Hierarchy Process.** The Analytic Hierarchy Process (AHP) is a decision analysis methodology developed by American operations researcher T.L. Saaty. The decision-making problem is analyzed by decomposing it into multiple levels and factors, employing pairwise comparisons to ascertain their significance, and constructing a judgment matrix. The multi-attribute decision analysis method, precisely the eigenvector approach, is utilized to evaluate factor weights based on the factors gathered through the ratio a_ij_ to form matrix A [23]. The qualitative indicators are assessed through pairwise comparisons. Subsequently, the weight values and overall rankings of the indicators at each level are established to assist in making design decisions.

**Thematic Analysis.** Braun and Clarke (2006,2017) characterized thematic analysis as a research methodology that identifies, analyzes, and interprets themes within qualitative data domains [24–25]. It is a versatile methodology that operates independently of any specific epistemological framework. Qualitative research seeks to furnish a comprehensive and nuanced description and elucidation of the research subject (Holloway & Wheeler, 2010; Smith et al., 2011) [26–27]. The primary operational process of the topic analysis method entails identifying and selecting issues. This procedure involves analyzing and classifying collected text or data to discern and comprehend specific topics or concerns. Based on the data, we establish preliminary coding and then explore potential topics. We then conduct an in-depth examination of the identified topic, clearly defining and appropriately naming it. All findings are compiled into a manuscript, serving as a comprehensive report of the analysis and highlighting the key insights and patterns discovered throughout the process. In addition, the manuscript will offer recommendations for further research and practical applications based on the identified topics.

**Shape Grammar.** Initially introduced by scholar George Stiny in 1975, it is articulated as a quaternion formula SG= (S, L, R, I), where S represents a finite set of shapes, L denotes a finite set of labels, and R signifies a finite set of inference rules. The original shape is represented by I [28]. Two inference rules are involved: generative (substitution, addition, and deletion) and derivative (scaling, copying, mirroring, miscutting, rotating, and Bézier curve transformations). Utilizing these inference rules enables the design process to shift its emphasis from merely the functional structure and culture of the product. It redirects attention to the interrelations among forms.

**Fuzzy comprehensive evaluation method.** In 1956, Professor L. A. Zadeh introduced the fuzzy comprehensive evaluation method. This approach is grounded in fuzzy mathematics, allowing for the transformation of qualitative assessments into quantitative evaluations. It employs fuzzy mathematics to conduct an overall evaluation of entities or objects influenced by multiple factors. [29] In the design field, designers commonly utilize the fuzzy comprehensive evaluation method to score and assess various design schemes, compare multiple options, and ultimately select the most favorable one.

### 3.2. The structure of the design process for Chizhou Nuo masks

The research on the extraction of cultural factors and design transformation of Chizhou Nuo masks is primarily conducted through five key aspects: First, field research is undertaken to gather and organize literature and information pertaining to Chizhou Nuo masks, alongside the creation of a distribution map of Chizhou Nuo culture. This involves analyzing and studying the cultural factors in their geographical context and elucidating their interrelationships. Second, factor analysis methodology is employed. The Chizhou Nuo mask culture is categorized into two primary classifications: dominant factors and recessive factors. This classification is based on the differentiation between explicit and implicit factors. Three dominant factors representative of Chizhou Nuo mask culture and one recessive factor are identified to create a factor mapping. Third, the Analytic Hierarchy Process is employed to objectively empower the Chizhou Nuo mask culture factors, ranking their significance to derive design factors. Subsequently, thematic analysis is conducted to gather data through semi-structured interviews, which are then analyzed to extract consumer demand indicators. Additionally, shape grammar is utilized to decompose and reorganize the design factors obtained from the evaluation results, culminating in the creation of a design product that aligns with contemporary aesthetic standards. Finally, using fuzzy comprehensive evaluation, three groups of design schemes were scored and assessed. The best scheme was selected, and further optimization was carried out on that scheme (S1 Fig).

## 4. Results

### 4.1. Collecting information on Chizhou Nuo masks

Initially, examining pertinent literature, governmental reports, and audiovisual materials. First, summarize and conclude the information to ascertain the distribution of the Nuo Chizhou geographical map; second, visit Nuo villages for photography and engage in dialogue with local villagers and Nuo opera practitioners to analyze the composition of Chizhou Nuo masks and their cultural factors; finally, compile and organize the collected information. Comprehensive research was conducted on both the tangible and intangible aspects of Chizhou Nuo opera, examining their interconnections and the factors hindering their dissemination in order to establish a framework for cultural research and analysis of Chizhou Nuo culture (S2 Fig).

### 4.2. Extracting cultural factors and modeling of Chizhou Nuo masks

The Chizhou Nuo masks exhibit both visible and tacit cultural differences. The dominant factor refers to the user’s direct visual observation of the product derived from the gene, including aspects such as morphology, color, and pattern. Conversely, the recessive factor is concealed within the cultural context, encompassing emotional connotations, folklore, symbolic semantics, and other elements, which require more in-depth exploration. The Chizhou Nuo masks will conceal the cultural expressions, importance, temporal context, and background of the ceremony, which will be synthesized and described accordingly [30]. This paper conducts a comprehensive analysis of Chizhou Nuo culture through literature review and field research, engaging in discussions and interviews with experts and scholars. The primary focus of the study is the Nuo mask culture, examining its morphology, color, pattern, and semantics as four cultural factors. Utilizing CorelDRAW X7 and Adobe Illustrator 2024 for picture creation and visual optimization throughout the construction of a hierarchical model for the extraction of cultural factors related to Chizhou Nuo masks (S3 Fig).

### 4.3. Extraction of cultural factors from Chizhou Nuo Masks

#### 4.3.1. Extraction of morphological factors.

The entirety of Nuo opera performance is inextricably linked to the Nuo mask. In the Chizhou Meijie region, Nuo masks are referred to as “face” or “Bodhi.” In Nuo performances, the mask represents the deities associated with the performer and serves as a symbol [31]. Chizhou Nuo masks emphasize the depiction of the facial characteristics of deities, effectively utilizing local facial feature distortions. The masks are intricately crafted, exhibiting unique shapes, a pronounced sense of form, and diverse, elaborate patterns. They are exaggerated, seemingly animated while maintaining a balanced, stylized, and harmonious unity, showcasing a profound rustic and rugged beauty. Chizhou Nuo masks, categorized by distinct performance repertoires, represent various character identities to fulfill diverse practical purposes and can be classified into divine, furious, and portrait types. Various Nuo masks can differentiate between distinct forms of Nuo opera, as the extraction of morphological characteristics is crucial. The morphological characteristics and outline optimization of Chizhou Nuo masks in line extraction. Effectively extracted example lines to illustrate the morphological characteristics of Chizhou Nuo masks (S1 Table).

#### 4.3.2. Extraction of color factors.

The Chizhou Nuo masks exhibit visual qualities, with color being the most prominent factor. The Chizhou Nuo mask features intricate patterns and vibrant colors, emphasizing the balance of the overall design. The patterns and colors are harmoniously combined, resulting in a cohesive appearance that is not cluttered. Masks typically feature red, yellow, black, green, and various other colors, generally limited to a maximum of three hues. A primary color usually constitutes the main body, complemented by additional colors as decorations, resulting in a striking overall color contrast that is aesthetically pleasing [32]. Effective color coordination enhances visual appeal for the audience and immerses them in the narrative, fostering a connection with the performers. The Chizhou Nuo masks are held by the inheritors, characterized by ingenious design, diverse variations, vibrant colors, and a lack of excess, being colorful yet refined. The classification and collecting of Chizhou Nuo mask colors serve as the foundation for identifying and extracting representative color factors, resulting in the establishment of the Chizhou Nuo mask color factor (S2 Table).

#### 4.3.3. Extraction of pattern factors.

The ornate pattern of the Chizhou Nuo mask is primarily evident in the facial features and headgear, designed to differentiate the identity and gender of the figures. The mask pattern factor features vibrant colors and intricate, comprehensive designs, encompassing various elements such as eyebrow patterns, eye motifs, head decorations, and floral and botanical patterns. Many of these patterns originate from individuals’ daily experiences; various forms of opera and living conditions impart distinct pattern attributes, serving as a unique medium for conveying emotions through diverse pattern elements, thereby facilitating the differentiation of masks and opera characters. The Chizhou Nuo masks exhibit the most distinctive and often utilized patterns for the extraction of characteristic lines, resulting in the creation of the Chizhou Nuo mask pattern factor mapping (S3 Table).

#### 4.3.4. Extraction of semantic factors.

Various masks possess distinct symbolic languages and convey diverse meanings, although they also reference the mask that conceals individuals’ aesthetic aspirations and profound cultural significance. Nuo masks, shaped by millennia of evolution and infused with Huizhou’s distinctive philosophies—Taoism, Confucianism, Buddhism, and others—exhibit pronounced local characteristics. They encapsulate the unique aesthetic sensibilities of Chizhou’s populace, showcasing artistic styles that range from abstract to realistic. Their myriad expressions symbolize various themes, including the warding off of malevolent spirits, the continuation of lineage, the invocation of good fortune, and the anticipation of abundant harvests. During the COVID-19 pandemic, these masks also embodied the concept of epidemic deterrence, instilling confidence in the community. Designers will comprehensively showcase the cultural characteristics of Chizhou Nuo masks by utilizing design tools and methodologies to explicitly communicate latent semantic elements through contemporary design (S4 Table).

### 4.4. Development and assessment of the cultural factor evaluation system for Chizhou Nuo masks

During the research process of design factors derived from the Chizhou Nuo mask cultural factor mapping, it is essential to consider the similarities and complexities among the cultural factors to effectively classify and organize the elements of the Chizhou Nuo mask. Furthermore, the Analytic Hierarchy Process can be computed using exact mathematical techniques. The cultural characteristics of Chizhou Nuo masks influence the significance of each level and will serve as a foundation for decision-making in design practice. This method enables researchers to evaluate the significance of each aspect more objectively, facilitating the creation of a more scientific and pragmatic design program.

The assessment framework for Chizhou Nuo mask cultural elements consists of four tiers: the target tier, the criterion tier, the index tier, and the evaluation factor tier. This study designates the target layer of Chizhou Nuo mask cultural characteristics as code A. The target layer signifies that the primary aim of our research is to examine all facets of Chizhou Nuo mask culture. In the criterion layer, dominant and recessive factors are designated as code B to clarify the evident and latent cultural elements considered in the study. The index layer comprises four specific cultural factors labeled as code C1, C2, C3, and C4. At the evaluation factor level, samples of cultural factors from each tier were extracted and assigned code D. This coding system facilitates the organization of content by each level, ensuring a structured and systematic investigation. This stratified research method enables a complete understanding and analysis of all facets of Chizhou Nuo mask culture, hence offering robust support for future research and preservation efforts. Construct the Chizhou Nuo mask culture factor assessment system model according to the criteria mentioned above (S4 Fig).

### 4.5. Design factor evaluation

This study employs the Chizhou Nuo mask cultural factor assessment model to ascertain the significance of each cultural factor. The Analytic Hierarchy Process is utilized to score various cultural factors at the same level, and weights are allocated based on the relative importance of the hierarchical factors. The judgment matrix was developed using the participants’ rating results, subsequently identifying the most typical cultural characteristics of Chizhou Nuo masks. Due to the varying evaluators for each aspect of perceived differences, a multi-level evaluation index system was constructed to calibrate test results and mitigate errors stemming from subjective personal factors, utilizing a nine-level scale in the questionnaire regarding the cultural factors of the Chizhou Nuo mask. In order to quantify the judgment matrix, the scaling method from 1to 9was used to assign weights to the indicators (scales and meanings are shown in S5 Table). Specialists from relevant disciplines established an evaluation committee to analyze the significance of the cultural characteristics associated with the Chizhou Nuo mask. The geometric mean approach was employed to derive a singular matrix from the collective input of various experts and to compute

The corresponding weights. This was succeeded by several rounds of discussions and summaries to ultimately derive the judgment matrices and the weight values for each indication in total target layer A and comprehensive assessment layer B, as presented in Tables 1 and 2.

**Table 1:** Layer Judgment Matrix and Weights for Layer A.

A	B1	B2
B1	1.000 0	1.123 3
B2	0.890 2	1.000 0
weight of a single layer	0.529 0	0.471 0

**Table 2:** Judgment Matrix and Weights for Layer B.

B1	C1	C2	C3	B2	C4
C1	1.000 0	0.331 6	3.515 1	C4	1
C2	3.015 7	1.000 0	6.000 4		
C3	0.284 5	0.166 7	1.000 0		
weight of a single layer	0.260 5	0.649 9	0.089 6	weight of a single layer	1


$$Wi=\frac{{\left(\Pi_{j=1}^{n}a_{ij}\right)}^{\frac{1}{n}}}{\sum {\mathrm{\backslash nolimits}}_{i=1}^{n}{\left(\Pi_{j=1}^{n}a_{ij}\right)}^{\frac{1}{n}}},i=1,2,3\ldots n$$ (1)

In practice, experts may reach divergent judgments when comparing two indicators; therefore, it is essential to evaluate the consistency of the generated judgment matrix to ensure the reasonableness of the indicator weights. Consequently, it is essential to evaluate the consistency of the judgment matrix developed regarding the cultural factors of the Chizhou Nuo mask.

(1) Calculate the consistency indicator CI with the following formula:

$$CI=\frac{\lambda_{\max}-n}{\left(n-1\right)}(\lambda_{\max}\;denotes\;the\;largest\;eigenvalue\;of\;the\;judgment\;matrix)$$ (2)

(2) The consistency indicator RI (RI is a constant value in Table 3 and is correlated with the quantity n);

**Table 3:** Judge the RI value of the average random consistency index of the matrix.

matrix rank	1	2	3	4	5	6	7	8	9	10	11	12
RI	0	0	0.52	0.89	1.12	1.26	1.36	1.41	1.46	1.49	1.52	1.54

The consistency ratio (CR) of the judgment matrix is computed; if CR is less than 0.1, the matrix is deemed acceptable and requires no adjustments. Conversely, if CR is greater than or equal to 0.1, the expert must revise the judgment matrix and repeat step 2 until CR is less than 0.1, as indicated by the subsequent formula.


$$CR=\frac{CI}{RI}$$ (3)

Through the independent analysis of Chizhou Nuo mask cultural factors, the consistency ratio (CR) of the judgment matrix for the total objective layer A and the comprehensive evaluation layer B is below 0.1. This result indicates that the judgment matrices for both layers exhibit consistency, allowing for an accurate assessment of the significance and comprehensive evaluation of these cultural factors. Furthermore, this provides a solid theoretical foundation for the continued research and promotion of Chizhou Nuo mask culture (Table 4).

**Table 4:** Indicators of consistency test for each factor layer.

norm	A	B1	B2	C1	C2	C3	C4
CI	0	0.018 0	0.000 0	0.070 7	0.012 0	0.038 0	0.040 7
RI	0	0.520 0	0.000 0	1.120 0	1.120 0	1.120 0	1.120 0
CR	0	0.034 7	0.000 0	0.063 1	0.010 7	0.033 9	0.036 3

The quantitative evaluation results indicate that the weight of B1 in B class surpasses that of B2, suggesting that the dominant factors of Chizhou Nuo masks hold greater significance in the design process, thereby implying that these factors are prioritized over others in the design of Chizhou Nuo masks. In the C layer, the weight of C2 is the most significant, indicating that the color element within the cultural aspects of Chizhou Nuo masks possesses representative traits that can resonate with the user. The color component is crucial in design, since it can express particular emotions and meanings that align with the user’s emotional requirements. The sorting results of the Chizhou Nuo mask culture factor D layer (Table 5) indicate that the color factor D21 received the highest weight, primarily associated with the veneration of deities; the morphology factor D15 also held significant weight, characterized by its rich diversity; the texture factor D32 was predominant in the texture layer, emphasizing the distinctiveness of the characters; when integrated with the semantic factor D45, these elements can serve as a representative of Chizhou Nuo mask cultural factors for design purposes. Design items can effectively convey the traditional significance of Chizhou Nuo masks while simultaneously addressing users’ multi-sensory requirements, embodying both inventive and practical attributes.

**Table 5:** Percentage of aggregated weights of cultural elements in Layer D of the Chizhou Nuo Mask.

**morphogenetic factor**	D11	0.044 9	**color factor**	D21	0.384 3
	D12	0.115 7		D22	0.241 1
	D13	0.063 9		D23	0.059 0
	D14	0.241 0		D24	0.060 4
	D15	0.534 6		D25	0.255 2
**pattern factor**	D31	0.283 5	**semantic factor**	D41	0.146 5
	D32	0.432 8		D42	0.079 0
	D33	0.1016		D43	0.434 6
	D34	0.141 5		D44	0.300 6
	D35	0.040 6		D45	0.039 2

### 4.6. User requirements assessment through thematic analysis

The thematic analysis method used in this study follows a clear set of steps: first, we look closely at the original data to understand its meaning and define the limits of each type by finding similar content and naming them, which helps us classify the data and explain the concepts. Second, we carried out the secondary theme coding based on the above results. By carefully reviewing and combining the organized data, we identified six main groups, moving from specific details to broader ideas. Finally, based on the second-level theme categories, the theme induction is further carried out to investigate the core categories that can control the overall situation so that the categories proposed in the early stage are centered on the core categories to achieve organic integration and deep integration, and form a theoretical system with internal logical association. Consequently, employing thematic analysis can facilitate the development and design of Chizhou Nuo’s cultural and creative products by digging, summarizing, and refining consumer demand, thereby ensuring that subsequent product designs align with market requirements.

#### 4.6.1. User requirements interviews.

This study employs a broad expert sample technique to guarantee the scientific, complete, and representative nature of primary data gathering, thereby obtaining multifaceted professional perspectives and experiences. This study comprises semi-structured interviews with ten specialists, including seasoned designers of cultural and creative items, academics in the discipline, experienced consumers, and sales professionals with extensive sales backgrounds. Semi-structured one-on-one interviews were performed with them, as outlined in S6 Table.

#### 4.6.2. Analysis of results.

Following the interviews, we employ Nvivo 12.0 to systematically code and label the gathered interview data meticulously, devoid of any researcher preconceptions or biases, by analyzing it word by word to formulate initial concepts and identify conceptual categories from the original data (S5 Fig). Employing thematic analysis to structure, evaluate, and condense the interview data, 46 preliminary categories were identified through open coding, encompassing a total of 328 nodes. The ten most frequently occurring categories included local symbol creativity, local characteristic narratives, local cultural products, enhancement of emotional experience, fusion of tradition and modernity, amalgamation of cultural connotations, visual appeal, marketing strategy, market demand orientation, and rational pricing strategy. Subsequently, secondary theme coding is conducted to derive six primary categories: local cultural features, product innovation, market adaptation, utility and practicality, product design, and recommendations for the advancement of cultural and creative industries. Ultimately, in addition to a secondary theme category, we proceed with further theme generalization; the objective of this phase is to identify the core category. This paper employs the tertiary theme to derive the two core main categories: the essential elements of cultural and creative product indicators and the deficiencies in cultural and creative product design. The affirmative key categories inform the ensuing design practice (S7 Table).

### 3.7. Transformation of cultural factors in Chizhou Nuo masks

In the design of Chizhou Nuo mask derivatives, the process begins by selecting the most representative morphogenetic, color, and pattern factors to define the visual subject’s external form, thereby providing users with an immediate visual experience. Subsequently, semantic factors are employed to augment the product’s cultural value, facilitating the preservation of cultural heritage. Ultimately, these elements are integrated to establish a design application system for Chizhou Nuo cultural derivatives, ensuring greater visibility for Chizhou Nuo culture.

Prioritize appealing and aesthetic design features in the selection process for the transformation design, enabling the audience to establish a connection with the culture of Chizhou Nuo through the product. The Chizhou Nuo mask culture factor weighting reveals that the most recognized element is factor D15, which serves as the foundational design form, optimizing user cognitive retention and evoking resonance with Chizhou Nuo mask culture. Factors D21 and D32 significantly influence color and pattern, effectively enhancing the product’s visual impact and aesthetic appeal. They can boost the product’s visual appeal, augment its perceived efficacy, and bolster its market competitiveness. Consequently, in product design, it is crucial to utilize D21 and D32 judiciously; while developing the altered semantic element D45, it must be managed meticulously to preserve the original meaning.

### 4.8. Chizhou Nuo mask design practice

To achieve design innovation, designers can employ generation rules inside shape grammar to modify the mask, preserving its original morphological characteristics but imparting new meaning and expression. Utilizing the cultural elements of the Chizhou Nuo mask, form factor D15 was chosen as the foundational design material. Designers employed Corel DRAW X7 to illustrate both the overall and intricate modeling of D15. Subsequently, modifications and adjustments were made to D15 to remove superfluous elements, preserving the defining characteristics of its lines to emphasize the mask’s overall shape and structure, thereby aligning it more closely with the design specifications. Alongside the morphological aspect, the pattern component is also a critical consideration in mask design. Throughout the design process, the designer may eliminate, refine, or rotate the pattern factor D31/D32 base unit visuals to create an entirely new derived pattern. These design processes can enrich the mask’s pattern, increasing its diversity and harmonizing with its overall shape, so strengthening its visual impact and cultural significance (S8 Table).

The conclusions of the thematic analysis of the interview text are integrated with the principles of contemporary visual product design and the aesthetic preferences of users, alongside the lifestyle habits of modern individuals. Using Chizhou Nuo mask graphic product design as a case study, the design practice employs the shape of grammatical design to incorporate extracted cultural factors into the translation process. This aligns with the principle of contemporary aesthetics, which involves executing a sequence of emoticon design projects. The D15 form factor serves as the foundation for the visual design pattern, embodying the essence of Nuo culture—upright, direct, and just. Its semantics resonate with the attitudes of contemporary youth, making the D15 an appropriate choice for design. This alignment facilitates the extension of Nuo cultural connotations, fostering cultural self-confidence among the youth. Secondly, regarding the form factor, the internal filling pattern factor D31/D32 established the primary design of the face, utilizing abstract processing techniques such as augmentation, subtraction, deformation, and further enhancement to amplify the graphic qualities of intimacy and charm. Utilizing the designated color factor D21 and optimization as the principal hue, select additional colors for a limited embellishment area that not only exemplify the characteristics of Nuo culture in Chizhou but also align with consumer preferences for aesthetically pleasing derivatives of Chizhou Nuo masks. The design mainly alters the extracted patterns in a localized way and produces three sets of IP images (S6 Fig).

### 4.9. Evaluation of schemes based on the fuzzy comprehensive evaluation method

In the design evaluation process, the fuzzy comprehensive evaluation (FCE) method helps tackle complicated problems that are hard to measure because of unclear personal opinions and many design factors [33]. In the subject analysis phase, interviewers were asked to evaluate three groups of IP image design ideas using the FCE method, which was paired with the evaluation hierarchy model based on the theme analysis (S7 Fig.).

The specific process for evaluating the design scheme using the fuzzy comprehensive evaluation method is as follows:

1. Establish a set of evaluation indicators.

Establish an evaluation index set. In this paper, the Chizhou Nuo Mask design index set V is divided into four standard layers for evaluation. Each standard layer index includes different sub-criteria layer indicators, V {A, B, C, D}. The factor set representing the sub-standard layer index is denoted as ai {a1, a2, a3,..., an} (i = 1, 2, 3).

2. Establish a fuzzy comprehensive evaluation weight set.

Taking the design evaluation model of the Chizhou Nuo mask as the research basis to determine the importance of each evaluation index, this study used the hierarchical analysis method in Chapter 3.5 for scoring. The relative weight was obtained by a hierarchical analysis scoring calculation and converted into the weight set (Table 6).

**Table 6:** Relative weights of each layer.

Criterion Layer	weight	Sub-criterion Layer	weight	Integration weights
A	0.293 3	A1	0.290 4	0.085 2
		A2	0.105 7	0.031 0
		A3	0.604 0	0.177 2
B	0.123 7	B1	0.350 8	0.043 4
		B2	0.096 2	0.011 9
		B3	0.553 0	0.068 4
C	0.525 7	C1	0.721 5	0.379 3
		C2	0.278 5	0.146 4
D	0.057 3	D1	0.700 1	0.040 1
		D2	0.299 9	0.017 2

3 The evaluation set consists of five levels: X {X1, X2, X3, X4, X5} = {very dissatisfied, dissatisfied, ordinary satisfied, satisfied, very satisfied}. The evaluation level in the evaluation set corresponds to the evaluation score range: 0–1, 1–2, 2–3, 3–4, and 4–5 (Table 7).

**Table 7:** Evaluation grade score interval table.

Evaluation indicators	Very satisfied	satisfied	Generally satisfied	dissatisfied	Very dissatisfied
Score range	(4-5]	(3-4]	(2-3]	(1-2]	[0-1]

4. Construction of the Fuzzy Comprehensive Evaluation Matrix. Invite expert evaluators to use the evaluation hierarchy model to assess three sets of IP image schemes. Calculate the frequency of scores given by evaluators to obtain the membership degree of each evaluation criterion at each evaluation level. Construct the fuzzy comprehensive evaluation matrix R for the design of cultural and creative products based on the Chizhou Nuo mask. Taking Scheme 1 as an example, R_A_ represents the cultural evaluation matrix of Criterion Layer 1; R_B_ represents the innovation evaluation matrix of Criterion Layer 1 provided by Party A; R_C_ represents the functional evaluation matrix of Criterion Layer 1; and R_D_ represents the market evaluation matrix of Criterion Layer 1.


$$R_{A}=\left[\begin{array}{ccccc} {}*20c0.9 & 0.1 & 0 & 0 & 0\\ 0.1 & 0.2 & 0.5 & 0.2 & 0\\ 0.1 & 0.1 & 0.4 & 0.3 & 0.1 \end{array}\right]$$ (4)

$$R_{B}=\left[\begin{array}{ccccc} {}*20c0 & 0 & 0.1 & 0.2 & 07\\ 0 & 0.1 & 0.3 & 0.4 & 0.2\\ 0.4 & 0.4 & 0.1 & 0.1 & 0 \end{array}\right]$$ (5)

$$R_{C}=\left[\begin{array}{ccccc} {}*20c0.9 & 0.1 & 0 & 0 & 0\\ 0.2 & 0.5 & 0.3 & 0 & 0 \end{array}\right]$$ (6)

$$R_{D}=\left[\begin{array}{ccccc} {}*20c0.3 & 0.4 & 0.1 & 0.2 & 0\\ 0.1 & 0.1 & 0.5 & 0.2 & 0.1 \end{array}\right]$$ (7)

5. A weighted average type of fuzzy operator is used to mathematically synthesize the weights of each evaluation indicator, which helps better integrate the information obtained from indicator weights and fuzzy comprehensive evaluation matrices. This process calculates the evaluation weight vector Z for each indicator in the standard layer of Design Scheme 1. The weight values for each indicator are derived from S13 Table:


$$W_{A}=\left[\begin{array}{ccc} {}*20c0.2904 & 0.1057 & 0.604 \end{array}\right]$$ (8)

$$W_{B}=\left[\begin{array}{ccc} {}*20c0.3508 & 0.0962 & 0.553 \end{array}\right]$$ (9)

$$W_{C}=\left[\begin{array}{cc} {}*20c0.7215 & 0.2785 \end{array}\right]$$ (10)

$$W_{D}=\left[\begin{array}{cc} {}*20c0.7001 & 0.2999 \end{array}\right]$$ (11)

Standard floor index weights for calculating the design scheme:


$$Z_{A}=F_{A}\times W_{A}=\left[\begin{array}{ccccc} {}*20c0.3323 & 0.1106 & 0.2944 & 0.2023 & 0.0604 \end{array}\right]$$ (12)

$$Z_{B}=F_{B}\times W_{B}=\left[\begin{array}{ccccc} {}*20c0.2212 & 0.2308 & 0.1192 & 0.1639 & 0.2648 \end{array}\right]$$ (13)

$$Z_{C}=F_{C}\times W_{C}=\left[\begin{array}{ccccc} {}*20c0.7051 & 0.2114 & 0.0836 & 0 & 0 \end{array}\right]$$ (14)

$$Z_{D}=F_{D}\times W_{D}=\left[\begin{array}{ccccc} {}*20c0.24 & 0.31 & 0.22 & 0.2 & 0.03 \end{array}\right]$$ (15)

6. On this basis, the membership degree of the fuzzy comprehensive evaluation index of the target layer is calculated:


$$Z_{V}=\left[\begin{array}{ccccc} {}*20c0.3323 & 0.1106 & 0.2944 & 0.2023 & 0.0604\\ 0.2212 & 0.2308 & 0.1192 & 0.1639 & 0.2648\\ 0.7051 & 0.2114 & 0.0836 & 0 & 0\\ 0.24 & 0.31 & 0.22 & 0.2 & 0.03 \end{array}\right]$$ (16)

$$W_{V}=\left[\begin{array}{cccc} {}*20c0.2933 & 0.1237 & 0.5257 & 0.0573 \end{array}\right]$$ (17)

$$Z=F_{V}\times W_{V}=\left[\begin{array}{ccccc} {}*20c0.5092 & 0.1899 & 0.1576 & 0.0911 & 0.0522 \end{array}\right]$$ (18)

7. Calculate the score of the design evaluation index of the cultural and creative products of Nuo masks in Chizhou:


$$Y=Z\times V=\left[\begin{array}{ccccc} {}*20c0.5092 & 0.1899 & 0.1576 & 0.0911 & 0.0522 \end{array}\right]\times \left[\begin{array}{c} {}*20c5\\ 4\\ 3\\ 2\\ 1 \end{array}\right]=4.01$$ (19)

The calculation results show that the overall evaluation of Design Scheme 1 for the Chizhou Nuo Mask cultural and creative product is very satisfactory. Similarly, Scheme 2 scored 3.1142, and Plan 3 scored 3.5241, both of which are satisfactory. We can conclude from the above scores that Scheme 1 is the optimal design scheme. (The calculation data for Schemes 2 and 3 are provided in the attachment S7.)

Subsequently, the evaluation results of Plan 1 were refined and redesigned. The evaluation results indicated that Plan 1 generally met consumer needs. Further analysis of the scores for each indicator in this plan revealed that “personal trend,” “cultural expansion,” and “online experience combined with offline purchases” scored low, suggesting that the design may need to better align with the preferences of contemporary young people. Therefore, the final plan added detailed elements to the entire IP and designed emoticons, primarily by altering local patterns to convey different emotions, aiming to attract young people’s attention to Nuo culture (S8 Fig).

## 5. Discussion

In the study of cultural and creative product design, numerous experts are increasingly focusing on the integration of cultural connotation and product functioning. Nevertheless, it has been observed that there are limited studies on the notion of industrial design. This study employed the AHP approach to evaluate, score, and rank the cultural characteristics of Chizhou Nuo masks in order to identify the design factors beforehand. The material is thoroughly analyzed using thematic analysis to identify consumer desires, ultimately applying shape grammar in design practice, finally, the fuzzy comprehensive evaluation is used to score and evaluate the design scheme, and the optimal scheme is obtained, and the design is further optimized. This study possesses specific limitations. Initially, there exists a notable deficiency in the research pertaining to the generalization of Chizhou Nuo cultural factors. For instance, the construction of the cultural gene pool is insufficiently comprehensive, and the generalization and classification criteria lack detail. Additionally, post-processing aspects require further examination. These areas should be prioritized for enhancement in subsequent studies. The sample size of the experts involved in the opinion interviews and evaluations is comparatively limited, resulting in certain constraints on the findings. In the subsequent study, the cohort of experts may be augmented, and other computational methodologies might be integrated to enhance the comprehensiveness of the research. The respondents were not meticulously categorized, and individuals from various areas or occupations may possess differing interpretations of consent items, thereby influencing the results produced. Consequently, this study may concentrate more on individuals from diverse geographic regions and other occupational categories in the future.

## 6. Conclusion

The design modification of Chizhou Nuo masks incorporates cultural elements, allowing the culture to possess a contemporary essence that can be preserved and evolved. This study utilizes memetic theory, a hierarchical model, thematic analysis, shape grammar, FCE and various research methodologies to develop the Chizhou Nuo mask cultural gene map and thoroughly investigate user needs, employing design examples to demonstrate the feasibility and validity of the Chizhou Nuo mask cultural gene pool. The integration of traditional culture with contemporary design methodologies facilitates comprehensive research and practice, enabling the accumulation of valuable insights and lessons for future design endeavors, thereby offering guidance and inspiration. Simultaneously, we may condense and disseminate the design outcomes to facilitate communication and collaboration with other designers and academics, thereby advancing the amalgamation of traditional culture and contemporary design evolution. The incorporation of Chizhou Nuo mask cultural elements in product design can embody the essence of Chinese culture while fulfilling user requirements, thereby establishing a connection between the public and Chizhou Nuo culture to promote a sustainable cultural legacy.

## Supporting information

S1 Fig Research Process. (TIF)

S2 Fig Thinking Framework for Analyzing Nuo Culture Research in Chizhou. (TIF)

S3 Fig Extracted Hierarchical Model of Chizhou Nuo Mask Cultural Factors. (TIF)

S1 Table Mapping of Morphological factors for Chizhou Nuo masks. (TIF)

S2 Table Color Factors Mapping of Chizhou Nuo masks. (TIF)

S3 Table Mapping of Pattern Factors for Chizhou Nuo Masks. (TIF)

S4 Table Mapping of Semantic Factors for Chizhou Nuo Masks. (TIF)

S4 Fig Model of Chizhou Nuo Mask Cultural Factor Assessment System. (TIF)

S5 Table Judgment matrix index importance level numerical scale table. (TIF)

S6 Table Outline for Semi-Structured Interviews Regarding Cultural and Creative Products of Chizhou Nuo Masks. (TIF)

S5 Fig Nvivo12-based word cloud of Chizhou Nuo cultural and creative products. (TIF)

S7 Table Translation process of interview data. (TIF)

S8 Table Progression of Product Design Elements for Chizhou Nuo Masks. (TIF)

S6 Fig The IP Image Design of Chizhou Nuo Mask. (TIF)

S7 Fig Chizhou Nuo Mask Design evaluation indicators hierarchical model. (TIF)

S8 Fig Optimized Design of the IP Image of the Chizhou Mask. (TIF)

S1 Appendix Criterrion Layer scoring data. (XLSX)

S2 Appendix Index Layer scoring data. (XLSX)

S3 Appendix Morphogenetic factor scoring data. (XLSX)

S4 Appendix Color factor scoring data. (XLSX)

S5 Appendix Pattern factor scoring data. (XLSX)

S6 Appendix Semantic factor scoring date. (XLSX)

S7 Appendix The calculation data for Schemes 2 and 3. (DOCX)

## References

[1] Ma GJ. A Comparative Study of Chinese Nuo Opera and East Asian Traditional Theater. [M]. Shanghai: Shanghai University Press; 2022.

[2] Meng FY. Masquerade and true love: An anthropological study of Nuo ritual music in Dangli, Yao Guichi, Anhui Province. Beijing: Culture and Art Publishing House. 2013.

[3] Ito S e i j i. Yízú cuōtàijí and Japanese New Year’s Customs. Differences, similarities, and exchanges between Chinese and Japanese folklore-Collection of academic papers on comparative studies of Chinese and Japanese folklore, Folk culture forum. 1991. p. 8–14.

[4] Oki Y. Report on a survey of Anshun Dixi opera. In: Tanaka I, editor. A comparative study of rural ritual drama in East Asia. Institute for Research in Oriental Cultures, University of Tokyo. 1992. p. 113–42.

[5] Hirota R. The Way of the Ghost: Masks and Rituals in China. Zhonghua Book Company. 2005.

[6] Suzuki M. Transmutation of Nations and Cultures in East Asia. Guizhou University Press. 2021.

[7] Inada A k i k o. Guizhou Anshun opera “singing book tune” and “performance tune”. In: Qu LY, Chen DX, editors. China Fanjingshan Nuo cultural symposium proceedings. China Theatre Press. 2004. p. 462–70.

[8] Oh S o o k y u n g. The self-selected collection of Chinese theater historians at home and abroad. Oh Soo Hyeong Volume. Elephant Press. 2018. p. 221–2.

[9] Choi YS, Cao Q. The structure and characteristics of Ping’an Nuo in Daochen Autonomous County, Guizhou Province, China. 2012 ed. Daochen Ancient N, editor. Guizhou Ethnic Publishing House. 2012.

[10] Wu Q, Hu HH, Zheng YY. Research on the analysis of color characteristics of Chizhou Nuo opera masks in the development of cultural and creative products. J Chizhou Univ. 2023;37(05):104–9. doi: 10.13420/j.cnki.jczu.2023.05.022

[11] Xiong AL. Research on stylistic features and color of Guizhou Nuo masks. China Academy of Art. 2013.

[12] Wang WW, Hu YK, Jin X. Research and application of traditional culture design element extraction model. Packaging Engineering. 2014;35(06):73-76 81. doi: 10.19554/j.cnki.1001-3563.2014.06.018

[13] Chen X, Liu Y. Extraction and design application of shadow culture factors based on satisfaction analysis. J Graphics. 2019;40(05):953–60.

[14] Gou BC, Yu H, Li ZF. Research on gene extraction and design application of half-slope colored pottery culture. Journal of Northwestern Polytechnical University (Social Science). 2011;31(04):66–9.

[15] Jiang F. Research on the extraction and design application of Hakka cultural factors in Gannan. Furniture Interior Design. 2021;(12):60–5. doi: 10.16771/j.cn43-1247/ts.2021.12.013

[16] Zhu Q, Rahman R, Alli H, Effendi RAARA. Souvenirs Development Related to Cultural Heritage: A Thematic Review. Sustainability. 2023;15(4):2918. doi: 10.3390/su15042918

[17] Cui X, Yang Y, Li SS. Mining and evolutionary analysis of intangible cultural heritage protection themes in China’s archives based on LDA model - a comparative perspective with the intangible cultural heritage protection center. Library Information Service. 2022;66(23):82–92. doi: 10.13266/j.issn.0252-3116.2022.23.009

[18] Lu AS, Green MC, Thompson D. Using Narrative Game Design to Increase Children’s Physical Activity: Exploratory Thematic Analysis. JMIR Serious Games. 2019;7(4):e16031. doi: 10.2196/16031 31750833 PMC6895869

[19] Liu M, Zhu X, Chen Y, Kong Q. Evaluation and design of dining room chair based on analytic hierarchy process (AHP) and fuzzy AHP. BioRes. 2023;18(2):2574–88. doi: 10.15376/biores.18.2.2574-2588

[20] Sun Y, Tao G, Xiong G, Chen H. The Fuzzy-AHP Evaluation Method for Unmanned Ground Vehicles. Appl Math Inf Sci. 2013;7(2):653–8. doi: 10.12785/amis/070232

[21] Dawkins R. The selfish gene. Oxford: Oxford University Press. 1976.

[22] Gao C, Li Y, Sun ML. Application of cultural symbols of collections in serialized product design. Packaging Engineering. 2017;38(4):47–50.

[23] Saaty TL. Analytic Hierarchy Process. Mathematical Models Decision Support. 2013;(4):109–21.

[24] Braun V, Clarke V. Using thematic analysis in psychology. Qualitative Res Psychol. 2006;3(2):77–101. doi: 10.1191/1478088706qp063oa

[25] Clarke V, Braun V. Thematic analysis. J Positive Psycholy. 2016;12(3):297–8. doi: 10.1080/17439760.2016.1262613

[26] Holloway I, Wheeler S. Qualitative research in nursing and healthcare. 3rd ed. Oxford, UK: Wiley-Blackwell. 2010.

[27] Smith J, Bekker H, Cheater F. Theoretical versus pragmatic design in qualitative research. Nurse Res. 2011;18(2):39–51. doi: 10.7748/nr2011.01.18.2.39.c8283 21319483

[28] Jia XN. Research on the styling design of small snow removal vehicle based on shape grammar. Beijing: Journal of China University of Mining & Technology. 2019.

[29] Yang DM, Zhang JN, Xu XY. Design of elderly shopping cart based on fuzzy comprehensive evaluation method. J Machine Design. 2016;33(9):117–20.

[30] Zhang AH, Xu K. Cultural gene inheritance and design transformation of southern dynasty stone carvings based on AHP-rooting theory. J Nanjing Univers Arts (Fine Arts & Design). 2023;(3):184–90.

[31] Lv GQ. Photography edited. Chinese witch Nuo civilization Nuo instrument Nuo customs Nuo dance Nuo opera. Hefei: Hefei University of Technology Press. 2019.

[32] Cheng YH. Guichi Nuo mask art and visual image design promotion. Art and Literature for the Masses: academic edition. 2016;:79–80.

[33] Liu Y, Fang P, Bian D, Zhang H, Wang S. Fuzzy comprehensive evaluation for the motion performance of autonomous underwater vehicles. Ocean Engineering. 2014;88:568–77. doi: 10.1016/j.oceaneng.2014.03.013

