# Supplementary figures and images for: Research on innovative design of Nuo mask based on Memetics-AHP-Thematic analysis-shape grammar
Source: PLoS One. 2025 Jul 14;20(7):e0326630. doi: 10.1371/journal.pone.0326630 (PMC12258567; doi:10.1371/journal.pone.0326630)

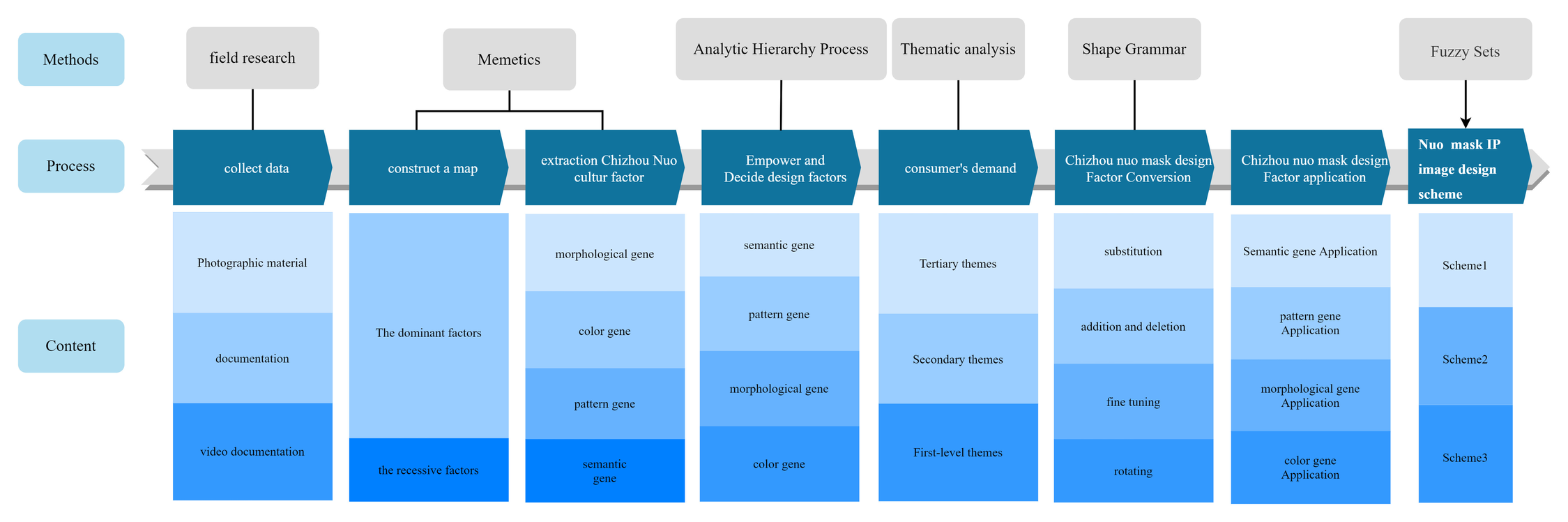

Supplement: S1 Fig — (TIF) [file pone.0326630.s001.tif]

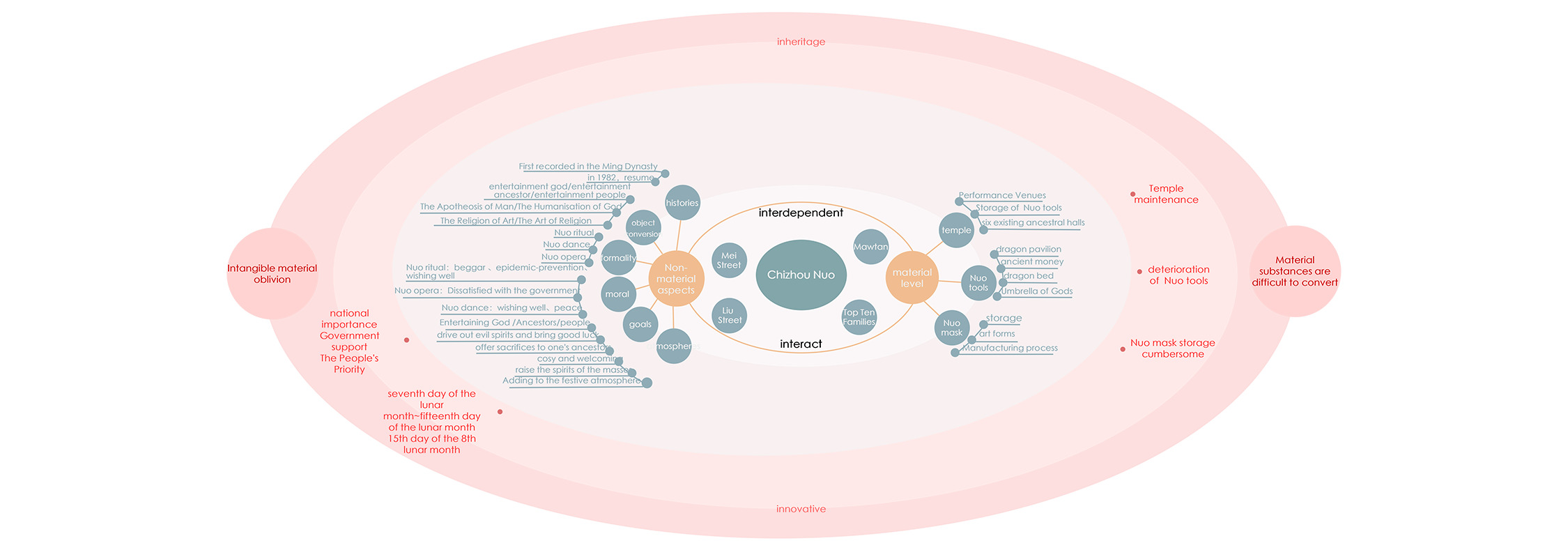

Supplement: S2 Fig — (TIF) [file pone.0326630.s002.tif]

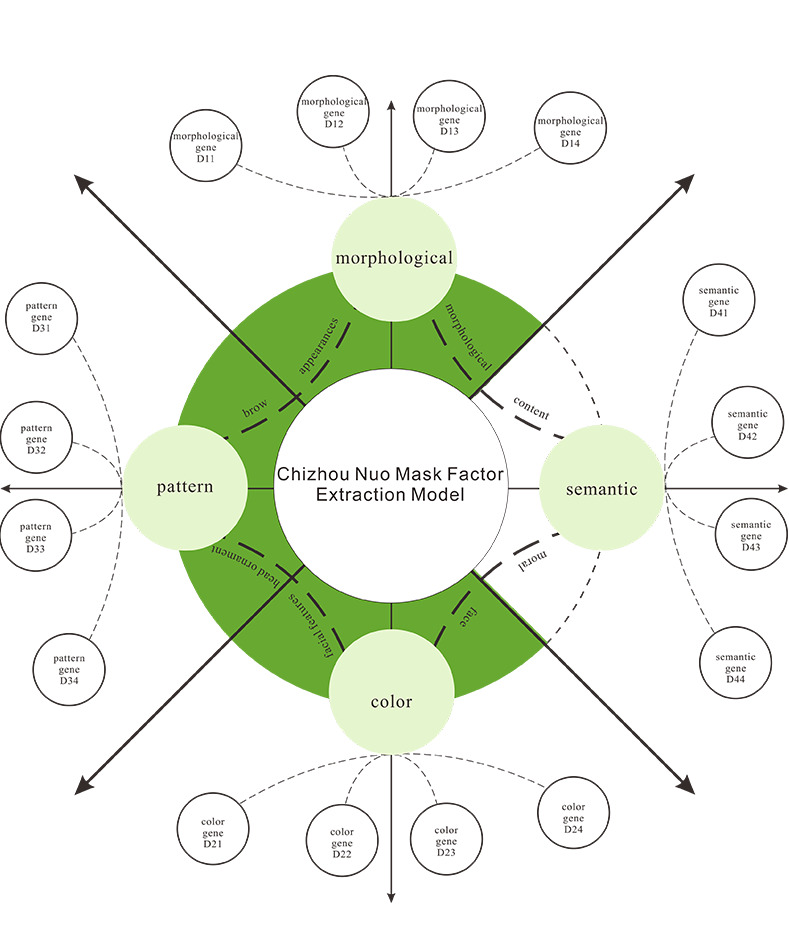

Supplement: S3 Fig — (TIF) [file pone.0326630.s003.tif]

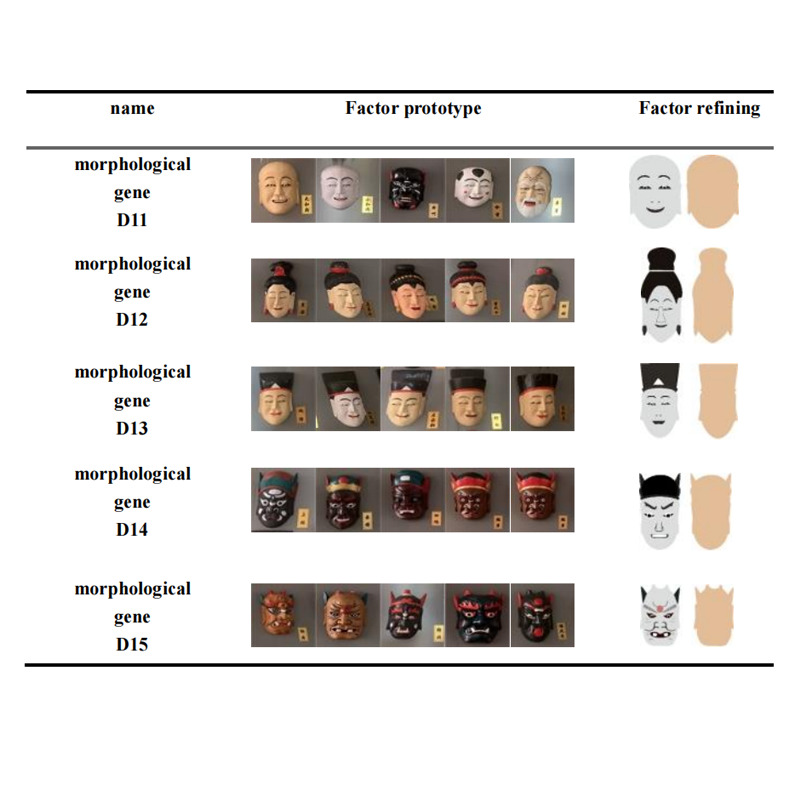

Supplement: S1 Table — (TIF) [file pone.0326630.s004.tif]

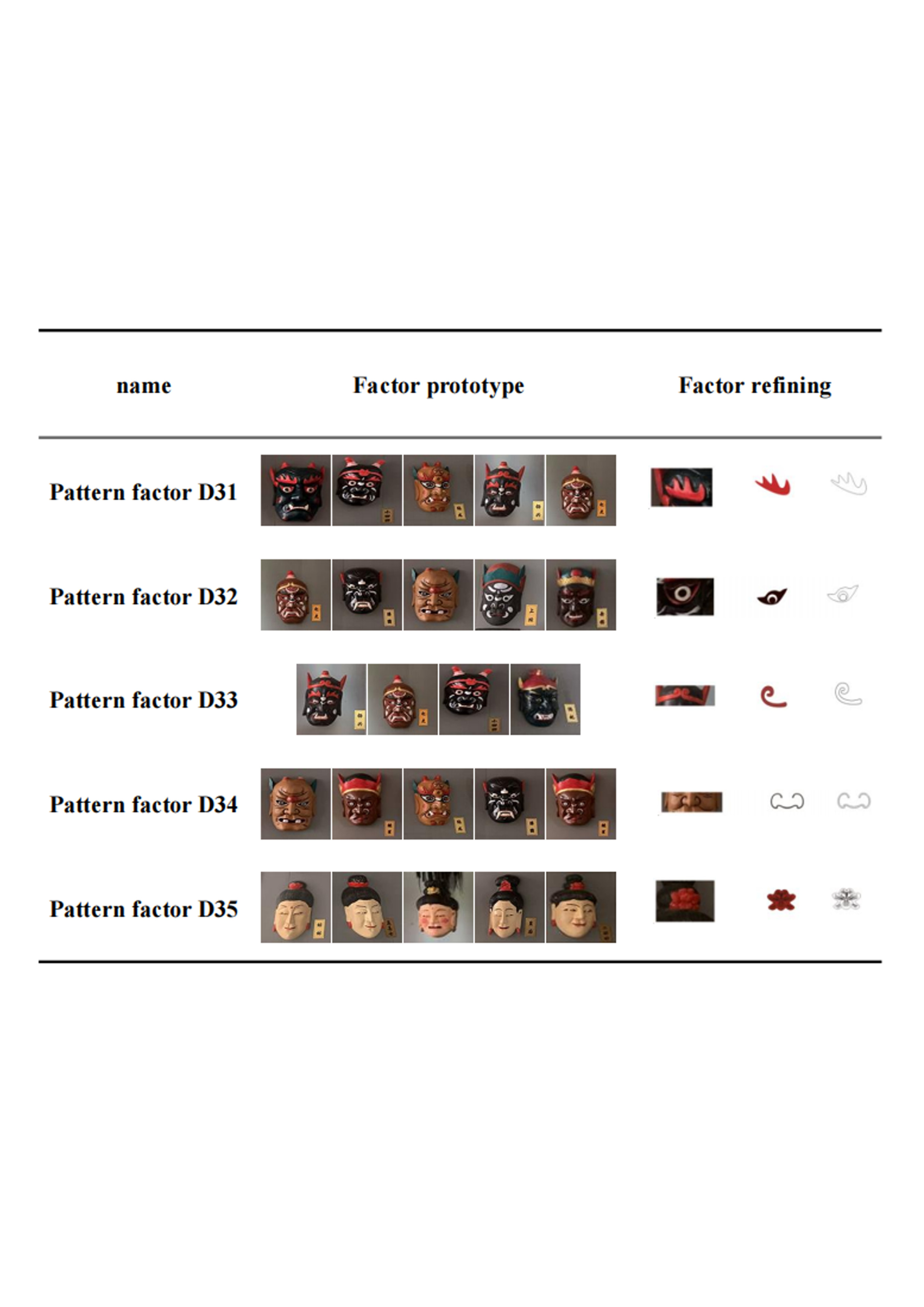

Supplement: S3 Table — (TIF) [file pone.0326630.s006.tif]

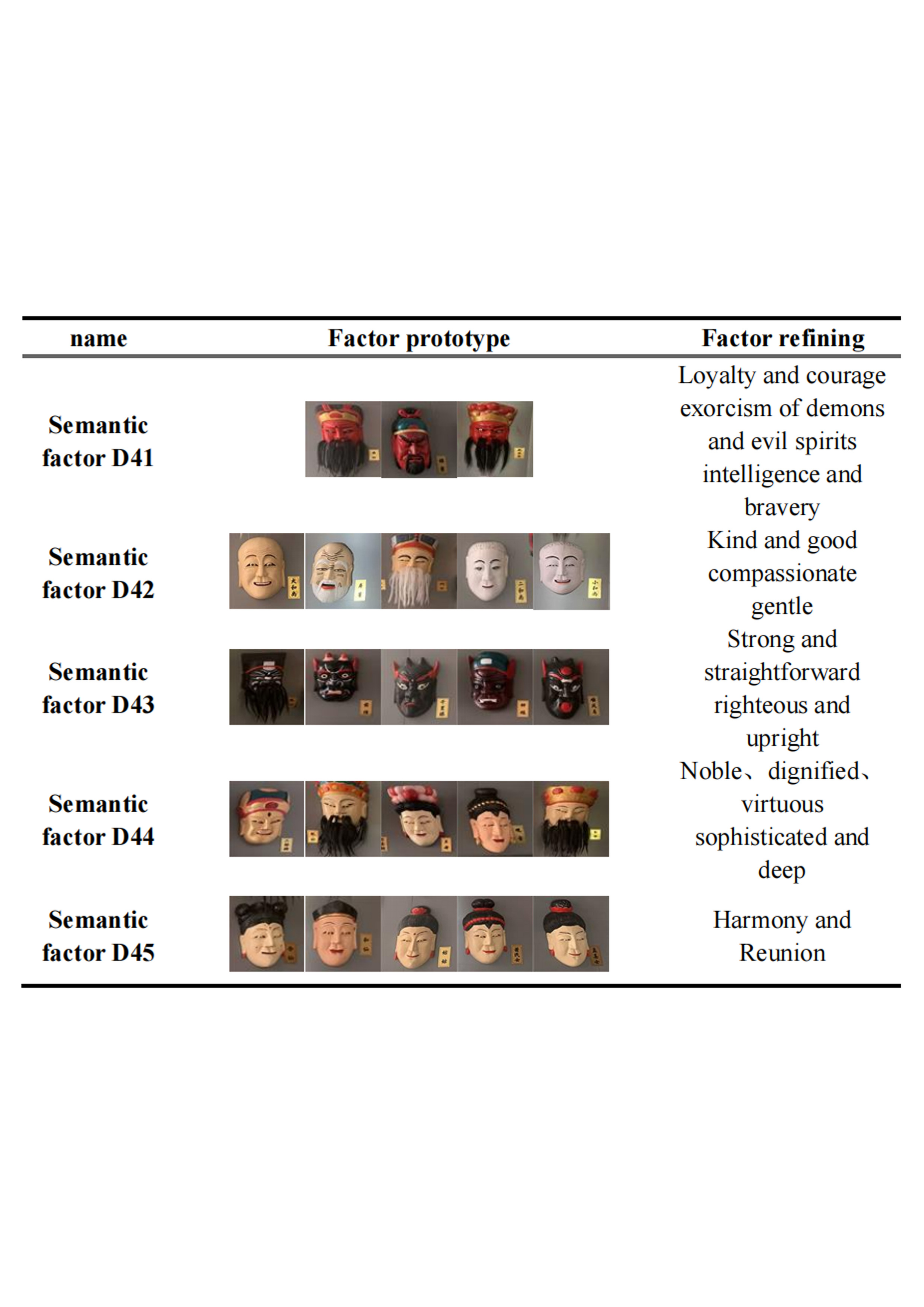

Supplement: S4 Table — (TIF) [file pone.0326630.s007.tif]

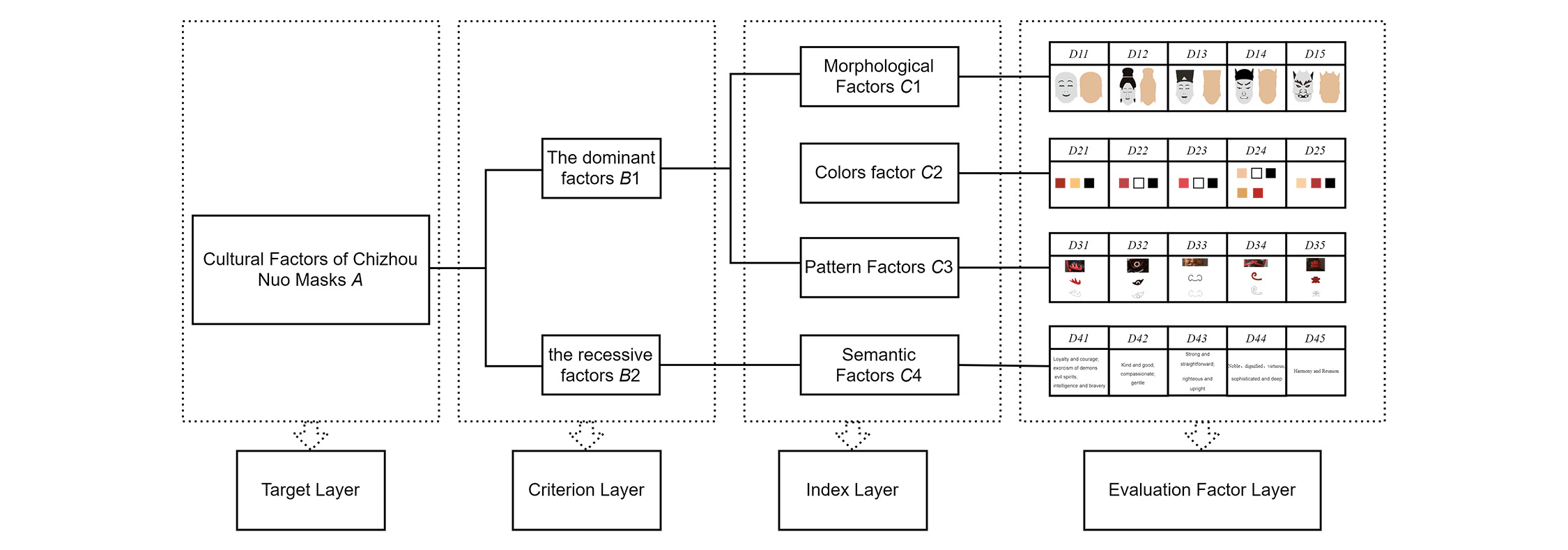

Supplement: S4 Fig — (TIF) [file pone.0326630.s008.tif]

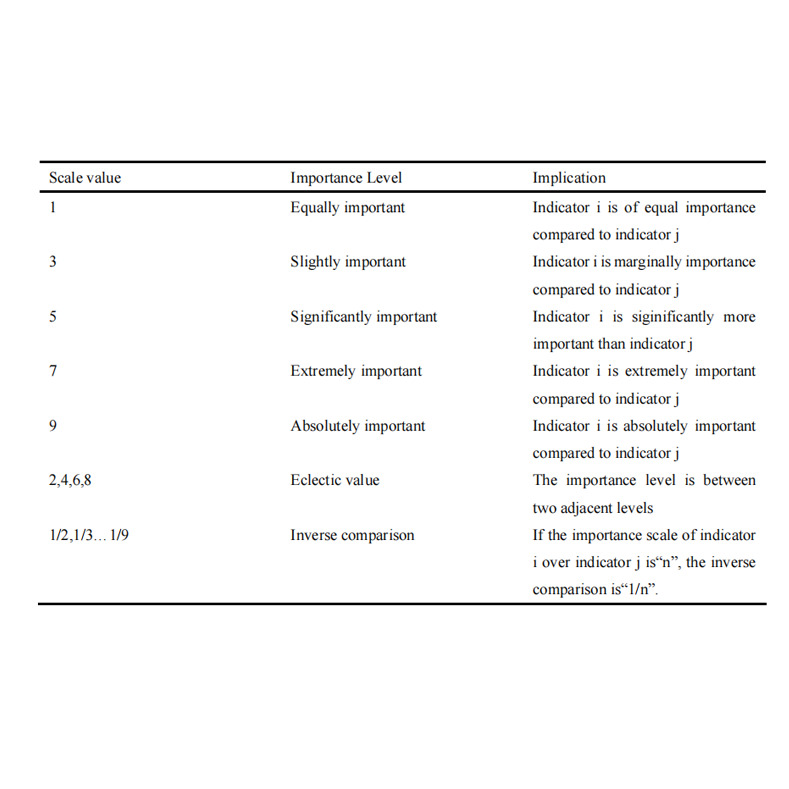

Supplement: S5 Table — (TIF) [file pone.0326630.s009.tif]

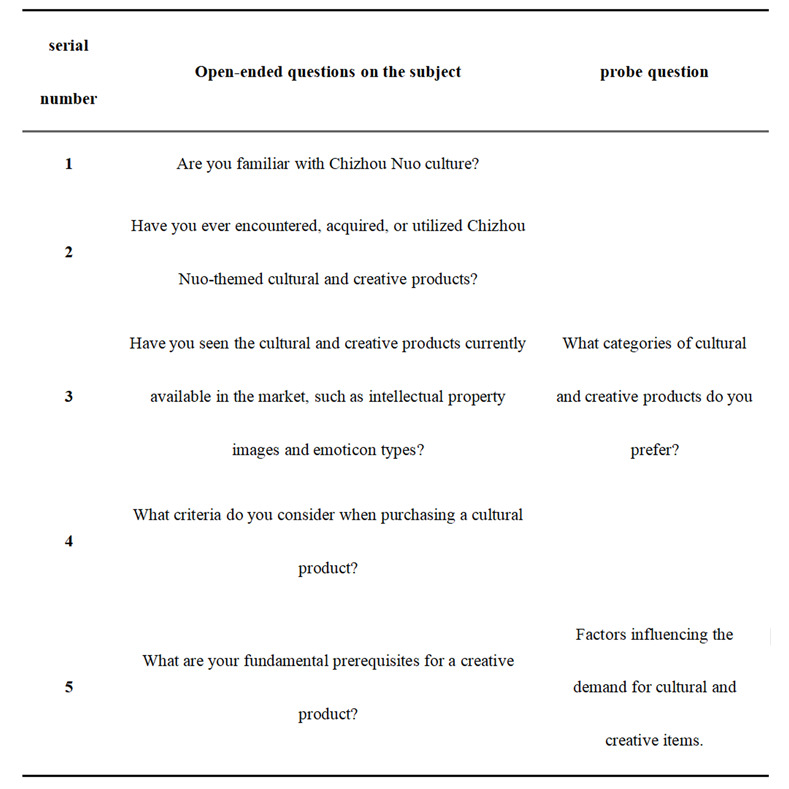

Supplement: S6 Table — (TIF) [file pone.0326630.s010.tif]

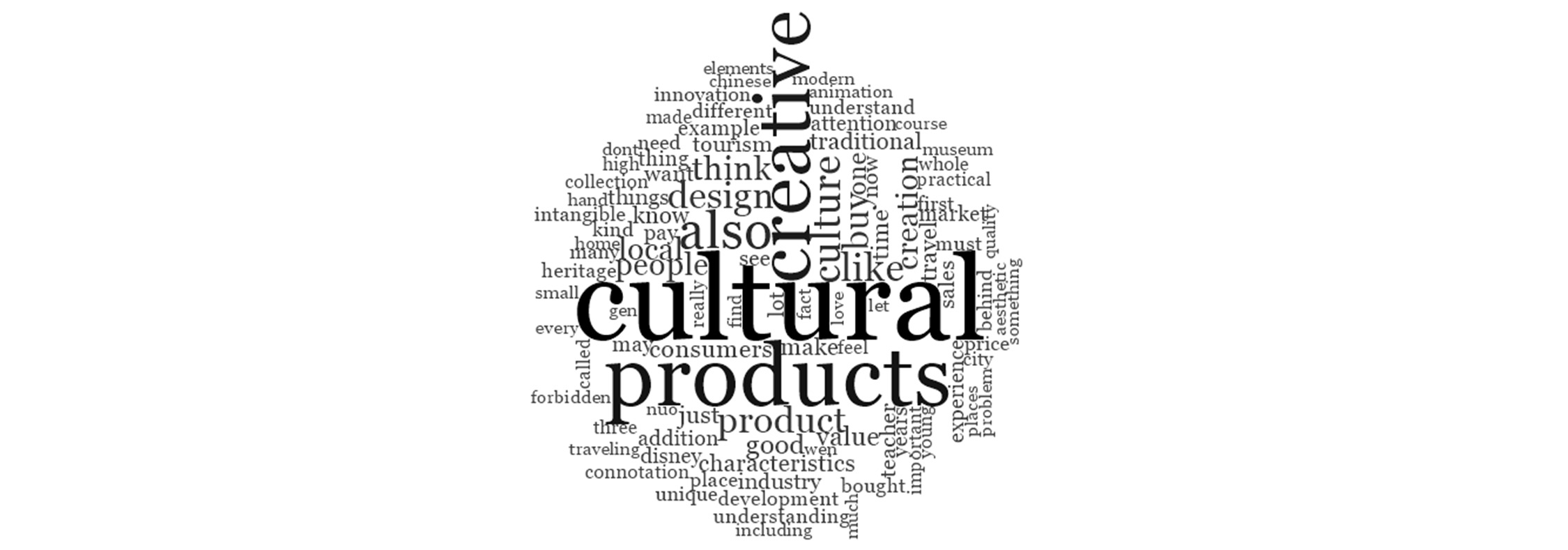

Supplement: S5 Fig — (TIF) [file pone.0326630.s011.tif]

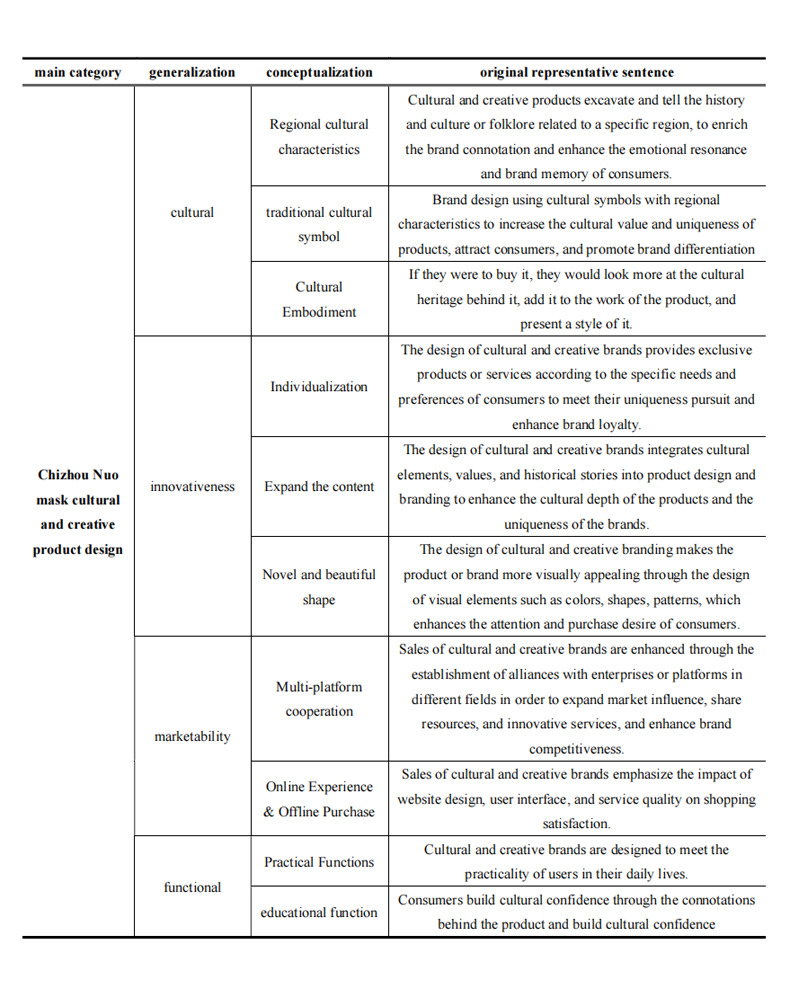

Supplement: S7 Table — (TIF) [file pone.0326630.s012.tif]

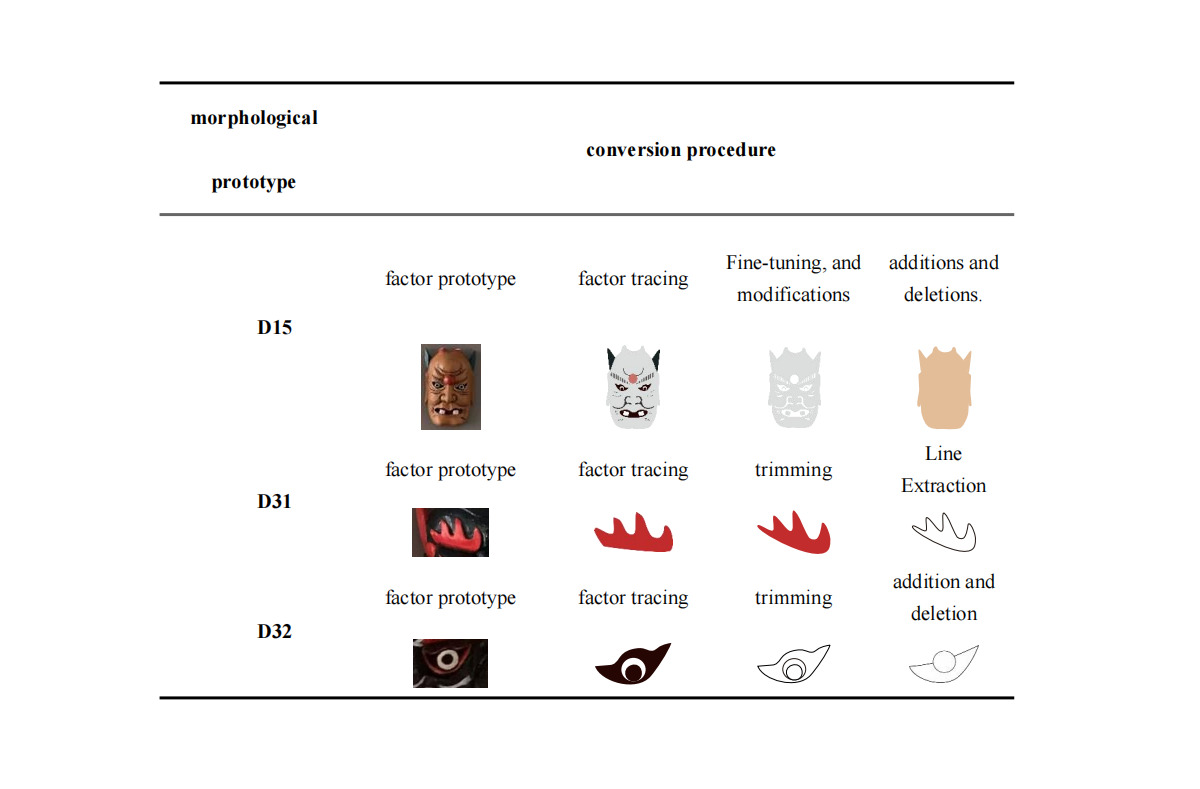

Supplement: S8 Table — (TIF) [file pone.0326630.s013.tif]

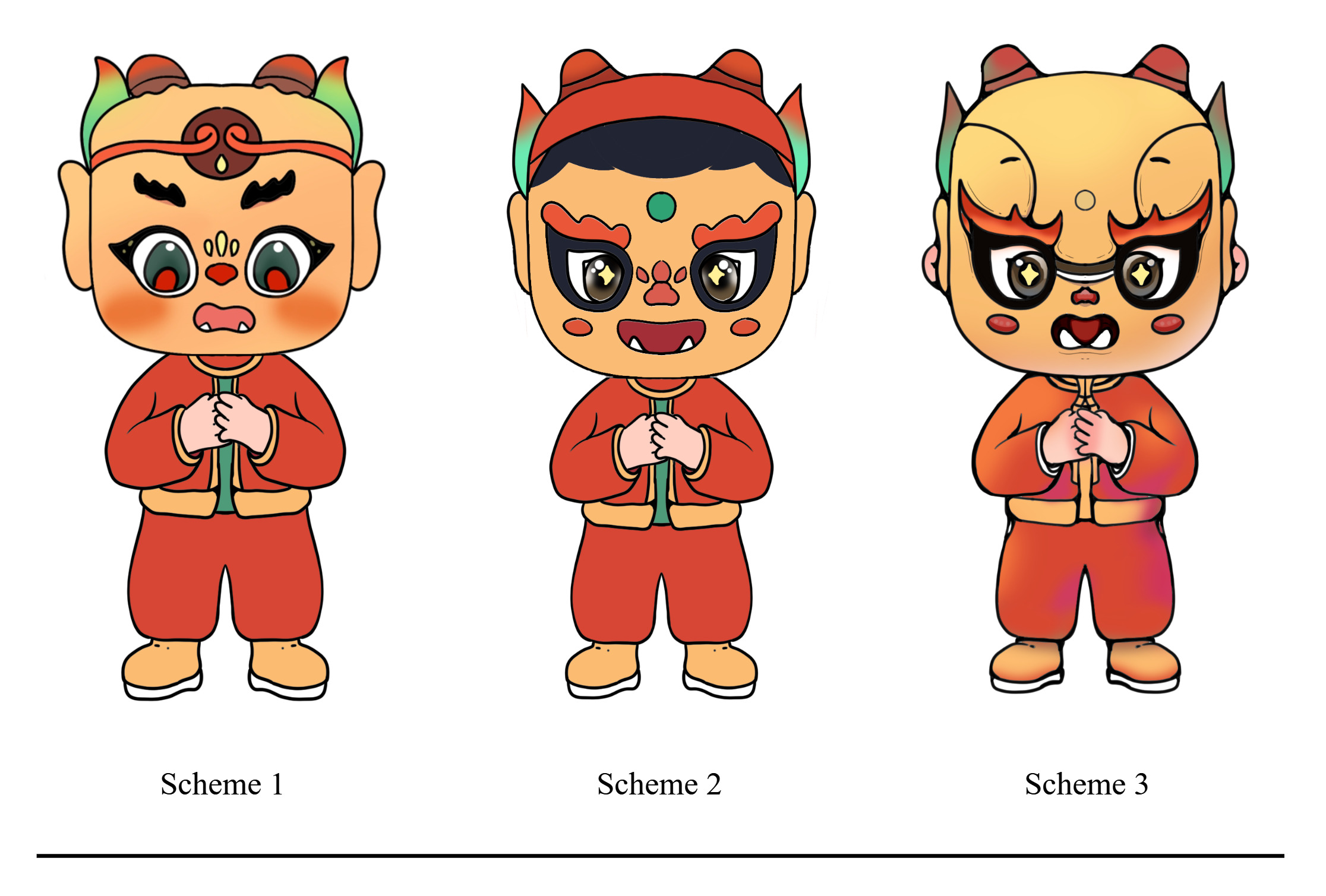

Supplement: S6 Fig — (TIF) [file pone.0326630.s014.tif]

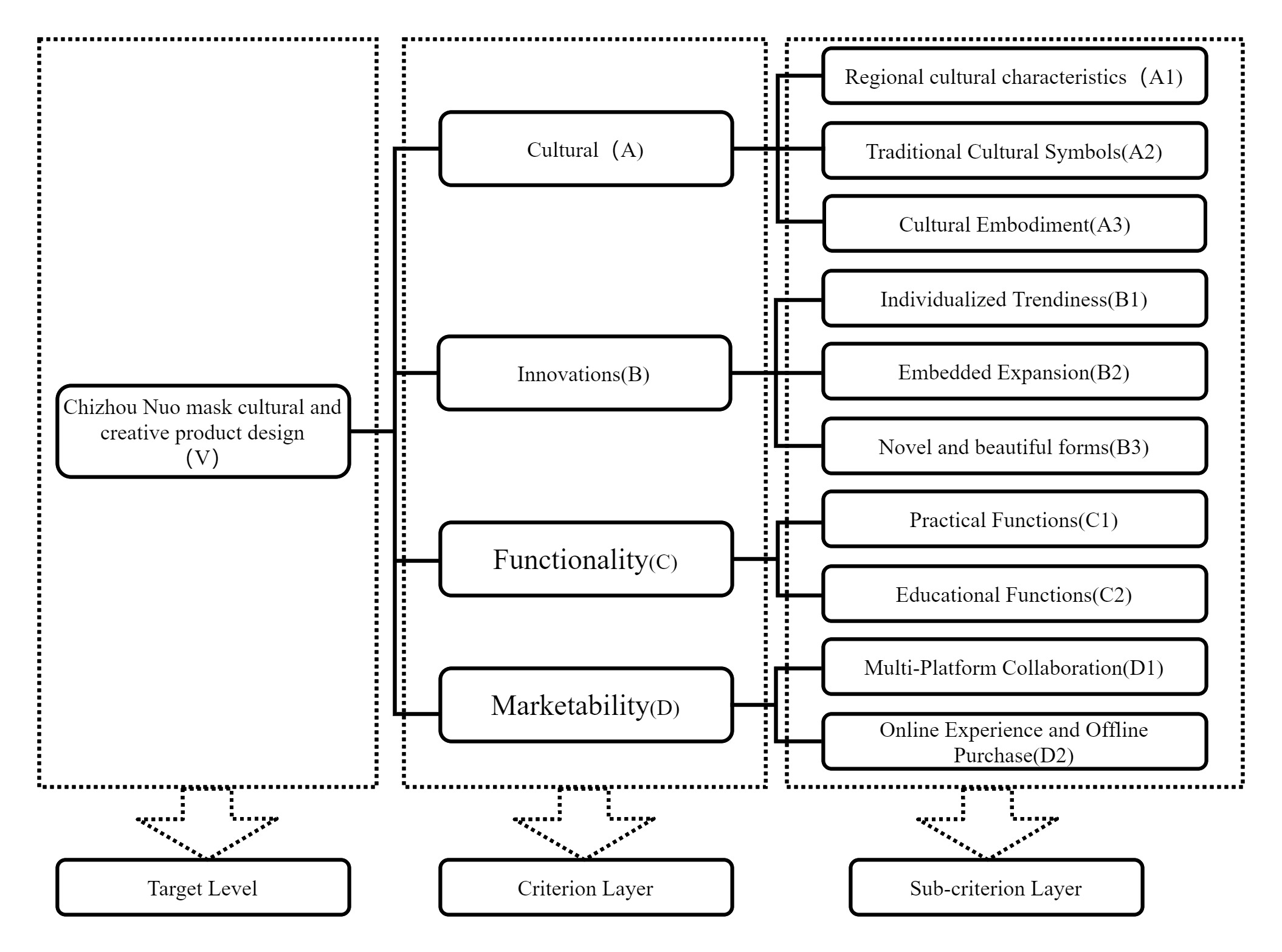

Supplement: S7 Fig — (TIF) [file pone.0326630.s015.tif]

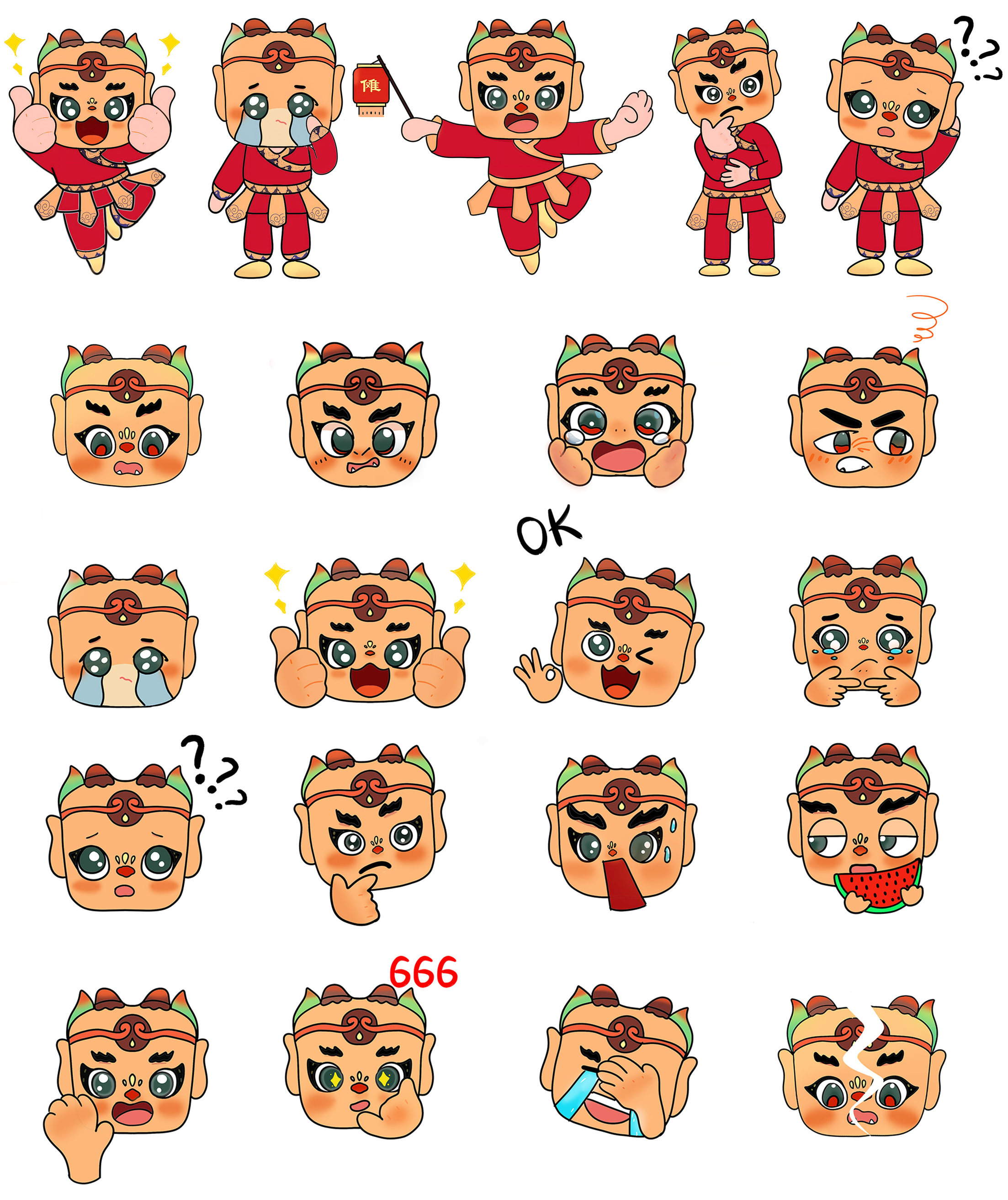

Supplement: S8 Fig — (TIF) [file pone.0326630.s016.tif]
