# Supplementary material for: Research on innovative design of Nuo mask based on Memetics-AHP-Thematic analysis-shape grammar
Source: PLoS One. 2025 Jul 14;20(7):e0326630. doi: 10.1371/journal.pone.0326630 (PMC12258567; doi:10.1371/journal.pone.0326630)
Supplement: S7 Appendix — (DOCX) [file pone.0326630.s023.docx]

**The following is the specific process of calculating the final evaluation scores of Schemes 2 and 3 in Chapters 3.10 (Fig. 6) through the fuzzy comprehensive evaluation method:**

**1. Scheme 2**

Count the number of times evaluators score each indicator in the sub-criterion layer, and obtain the degree of membership of each evaluation indicator relative to each evaluation level. Thus, the fuzzy comprehensive evaluation matrix R of each indicator of Nuo mask design scheme 2 is constructed.
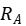
represents the evaluation matrix of the criterion layer Cultural for Scheme 2;
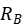
represents the evaluation matrix of the criterion layer innovations for Scheme 2;
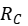
represents the evaluation matrix of the criterion layer functionality for Scheme 2; and
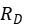
represents the evaluation matrix of the criterion layer marketability for Scheme 2:

$$R_{A}=\left[\begin{matrix} 0 & 0 & 0.4 & 0.3 & 0.3 \\ 0 & 0.3 & 0.5 & 0.2 & 0 \\ 0 & 0.3 & 0.4 & 0.3 & 0 \end{matrix}\right]$$

$$R_{B}=\left[\begin{matrix} 0 & 0.5 & 0.3 & 0.2 & 0 \\ 0 & 0.4 & 0.3 & 0.3 & 0 \\ 0 & 0 & 0.3 & 0.3 & 0.4 \end{matrix}\right]$$

$$R_{C}=\left[\begin{matrix} 0 & 0.3 & 0.4 & 0.3 & 0 \\ 0 & 0.3 & 0.3 & 0.4 & 0 \end{matrix}\right]$$

$$R_{D}=\left[\begin{matrix} 0 & 0.4 & 0.3 & 0.3 & 0 \\ 0 & 0.3 & 0.4 & 0.3 & 0 \end{matrix}\right]$$

Using a weighted average type fuzzy operator to synthesize the weights of each indicator with their corresponding evaluation matrix R, the evaluation weight vectors P for each indicator in the criterion layer of Scheme 2 is calculated.

According to Tables 13, the weight values of the indicators were obtained:

$$W_{A}=\left(\begin{matrix} 0.2904 & 0.1057 & 0.604 \end{matrix}\right)$$

$$W_{B}=\left(\begin{matrix} 0.3508 & 0.0962 & 0.553 \end{matrix}\right)$$

$$W_{C}=\left(\begin{matrix} 0.7215 & 0.2785 \end{matrix}\right)$$

$$W_{D}=\left(\begin{matrix} 0.7001 & 0.2999 \end{matrix}\right)$$

The weight vectors of the criterion layer indicators of the design scheme 2 can be calculated:

$$Z_{A}=F_{A}\times W_{A}=\left[\begin{matrix} 0 & 0.2127 & 0.4581 & 0.3051 & 0.0241 \end{matrix}\right]$$

$$Z_{B}\times W_{B}=\left[\begin{matrix} 0 & 0.2114 & 0.3267 & 0.2728 & 0.2221 \end{matrix}\right]$$

$$Z_{C}\times W_{C}=\left[\begin{matrix} 0 & 0.2997 & 0.3718 & 0.3285 & 0 \end{matrix}\right]$$

$$Z_{D}\times W_{D}=\left[\begin{matrix} 0 & 0.3697 & 0.3697 & 0.2606 & 0 \end{matrix}\right]$$

On this basis, the fuzzy comprehensive evaluation matrix for the target layer can be constructed:

$$Y=Z\times W=\left[\begin{matrix} 0 & 0.2127 & 0.4581 & 0.3051 & 0.0241 \\ 0 & 0.2114 & 0.3267 & 0.2728 & 0.2221 \\ 0 & 0.2997 & 0.3718 & 0.3285 & 0 \\ 0 & 0.3697 & 0.3697 & 0.2606 & 0 \end{matrix}\right]\times\left[\begin{matrix} 0.2933 \\ 0.1237 \\ 0.5257 \\ 0.0573 \end{matrix}\right]=\left[\begin{matrix} 0 \\ 0.2557 \\ 0.3997 \\ 0.3193 \\ 0.0253 \end{matrix}\right]$$

From the above, the comprehensive evaluation vector of Nuo mask design scheme 2 can be obtained:

$$Y=Z\times V=\left[\begin{matrix} 0 & 0.2557 & 0.3997 & 0.3193 & 0.0253 \end{matrix}\right]\times\left[\begin{matrix} 5 \\ 4 \\ 3 \\ 2 \\ 1 \end{matrix}\right]=3.1142$$

**2. Scheme 3**

Count the number of times evaluators score each indicator in the sub-criterion layer, and obtain the degree of membership of each evaluation indicator relative to each evaluation level. Thus, the fuzzy comprehensive evaluation matrix R of each indicator of Nuo mask design scheme 3 is constructed.
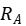
represents the evaluation matrix of the criterion layer Cultural for Scheme 3;
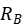
represents the evaluation matrix of the criterion layer innovations for Scheme 3;
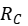
represents the evaluation matrix of the criterion layer functionality for Scheme 3; and
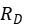
represents the evaluation matrix of the criterion layer marketability for Scheme 3:

$$R_{A}=\left[\begin{matrix} 0 & 0 & 0.1 & 0.2 & 0.7 \\ 0 & 0.2 & 0.3 & 0.5 & 0 \\ 0 & 0.1 & 0.3 & 0.6 & 0 \end{matrix}\right]$$

$$R_{B}=\left[\begin{matrix} 0 & 0.4 & 0.4 & 0.2 & 0 \\ 0 & 0.3 & 0.4 & 0.3 & 0 \\ 0 & 0 & 0.1 & 0.1 & 0.8 \end{matrix}\right]$$

$$R_{C}=\left[\begin{matrix} 0 & 0.1 & 0.3 & 0.6 & 0 \\ 0 & 0.1 & 0.2 & 0.7 & 0 \end{matrix}\right]$$

$$R_{D}=\left[\begin{matrix} 0 & 0.3 & 0.4 & 0.3 & 0 \\ 0 & 0.2 & 0.4 & 0.4 & 0 \end{matrix}\right]$$

Using a weighted average type fuzzy operator to synthesize the weights of each indicator with their corresponding evaluation matrix R, the evaluation weight vectors P for each indicator in the criterion layer of Scheme 3 is calculated.

According to Tables 13, the weight values of the indicators were obtained:

$$W_{A}=\left(\begin{matrix} 0.2904 & 0.1057 & 0.604 \end{matrix}\right)$$

$$W_{B}=\left(\begin{matrix} 0.3508 & 0.0962 & 0.553 \end{matrix}\right)$$

$$W_{C}=\left(\begin{matrix} 0.7215 & 0.2785 \end{matrix}\right)$$

$$W_{D}=\left(\begin{matrix} 0.7001 & 0.2999 \end{matrix}\right)$$

The weight vectors of the criterion layer indicators of the design scheme 3 can be calculated:

$$Z_{A}=F_{A}\times W_{A}=\left[\begin{matrix} 0 & 0.0814 & 0.2307 & 0.4463 & 0.2016 \end{matrix}\right]$$

$$Z_{B}\times W_{B}=\left[\begin{matrix} 0 & 0.1568 & 0.2134 & 0.1238 & 0.446 \end{matrix}\right]$$

$$Z_{C}\times W_{C}=\left[\begin{matrix} 0 & 0.0999 & 0.2719 & 0.6282 & 0 \end{matrix}\right]$$

$$Z_{D}\times W_{D}=\left[\begin{matrix} 0 & 0.2698 & 0.4796 & 0.3896 & 0 \end{matrix}\right]$$

On this basis, the fuzzy comprehensive evaluation matrix for the target layer can be constructed:

$$Y=Z\times W=\left[\begin{matrix} 0 & 0.0814 & 0.2307 & 0.4463 & 0.2016 \\ 0 & 0.1568 & 0.2134 & 0.1238 & 0.446 \\ 0 & 0.0999 & 0.2719 & 0.6282 & 0 \\ 0 & 0.2698 & 0.4796 & 0.3896 & 0 \end{matrix}\right]\times\left[\begin{matrix} 0.2933 \\ 0.1237 \\ 0.5257 \\ 0.0573 \end{matrix}\right]=\left[\begin{matrix} 0 \\ 0.1034 \\ 0.2617 \\ 0.4773 \\ 0.1246 \end{matrix}\right]$$

From the above, the comprehensive evaluation vector of Nuo mask design scheme 3 can be obtained:

$$Y=Z\times V=\left[\begin{matrix} 0 & 0.1034 & 0.2617 & 0.4773 & 0.1246 \end{matrix}\right]\times\left[\begin{matrix} 5 \\ 4 \\ 3 \\ 2 \\ 1 \end{matrix}\right]=3.5241$$
